# Supplementary material for: The causal relationship between the human gut microbiota and pyogenic arthritis: a Mendelian randomization study
Source: Front Cell Infect Microbiol. 2024 Nov 26;14:1452480. doi: 10.3389/fcimb.2024.1452480 (PMC11629706; doi:10.3389/fcimb.2024.1452480)
Supplement: Supplementary file 1 [file DataSheet1.zip › mendelian test/ebi-a-GCST90027737.csv_leave_one_out.pdf]

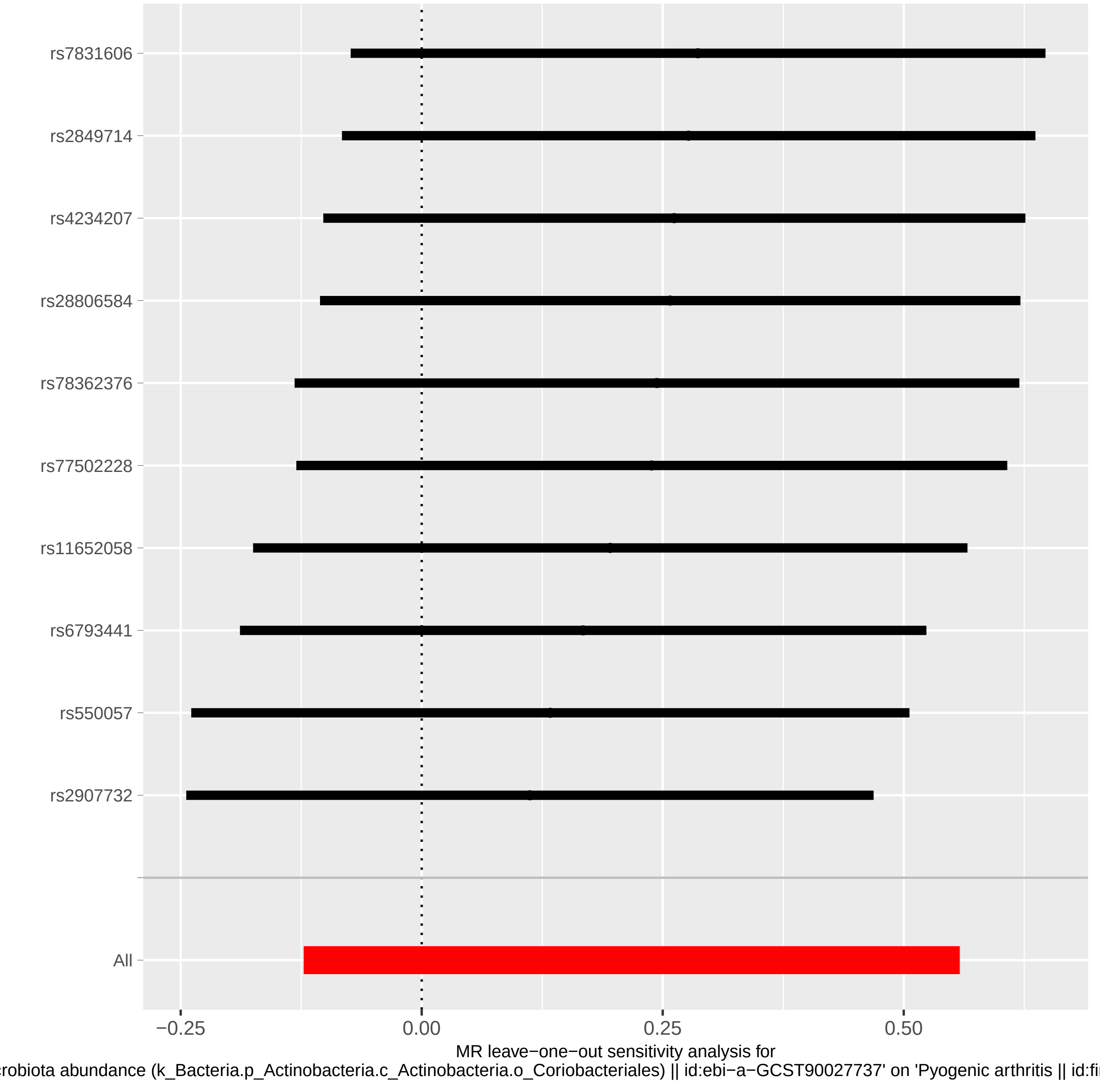
rs7831606
rs2849714
rs4234207
rs28806584
rs78362376
rs77502228
rs11652058
rs6793441
rs550057
rs2907732
All
−0.25
0.00
0.25
0.50
MR leave−one−out sensitivity analysis for
crobiota abundance (k_Bacteria.p_Actinobacteria.c_Actinobacteria.o_Coriobacteriales) || id:ebi−a−GCST90027737' on 'Pyogenic arthritis || id:fi
